# The Impact of War in Yemen on Immunization Coverage of Children Under One Year of Age: Descriptive Study

**DOI:** 10.2196/14461

**Published:** 2019-10-23

**Authors:** Amr Torbosh, Mohammed Abdulla Al Amad, Abdulwahed Al Serouri, Yousef Khader

**Affiliations:** 1 Yemen Field Epidemiology Training Program The Eastern Mediterranean Public Health Network Sanaa Yemen; 2 Department of Community Medicine, Public Health and Family Medicine Faculty of Medicine Jordan University of Science & Technology Amman Jordan

**Keywords:** immunization coverage, 2015 war, impact, Y-FETP, Yemen

## Abstract

**Background:**

After 2 years of war that crippled the capacity of the Yemeni National Health System and left only 45% of health facilities functioning, Yemen faced increasing vaccine-preventable disease (VPD) outbreaks and may be at high risk of polio importation.

**Objective:**

The aim of this study was to determine the impact of the 2015 war on the immunization coverage of children under 1 year.

**Methods:**

Data on vaccination coverage for 2012-2015 were obtained from the national Expanded Program on Immunization (EPI). The vaccination coverage was calculated at the national and governorate levels by dividing the number of actually vaccinated children by the estimated population of children under 1 year.

**Results:**

Although there was an increase from 2012 to 2014 in the national coverage for penta-3 vaccine (82% in 2012 vs 88% in 2014) and measles vaccine (70% in 2012 vs 75% in 2014), the coverage was still below the national target (≥95%). Furthermore, the year 2015 witnessed a marked drop in the national coverage compared with 2014 for the measles vaccine (66% in 2015 vs 75% in 2014), but a slight drop in penta-3 vaccine coverage (84% in 2015 vs 88% in 2014). Bacillus Calmette–Guérin vaccine also showed a marked drop from 73% in 2014 to 49% in 2015. These reductions were more marked in governorates that witnessed armed confrontations (eg, Taiz, Lahj, and Sa’dah governorates). On the other hand, governorates that did not witness armed confrontations showed an increase in coverage (eg, Raymah and Ibb), owing to an increase in their population because of displacement from less secure and confrontation-prone governorates.

**Conclusions:**

This analysis demonstrated the marked negative impact of the 2015 war on immunization coverage, especially in the governorates that witnessed armed confrontations. This could put Yemen at more risk of VPD outbreaks and polio importation. Besides the ongoing efforts to stop the Yemeni war, strategies for more innovative vaccine delivery or provision and fulfilling the increasing demands are needed, especially in governorates with confrontations. Enhancing EPI performance through supportable investments in infrastructure that was destroyed by the war and providing decentralized funds are a prerequisite.

## Introduction

### Background

Vaccination, one of the greatest achievements in medicine and public health, has greatly reduced morbidity, mortality, and health care costs [1]. Although many developing countries have seen a major reduction in vaccine-preventable diseases (VPD) owing to sustained use of vaccines, vaccination has not reached its full potential and at least 2 million people die every year from VPD [2]. Therefore, for vaccination programs to be effective, high rates of coverage must be maintained.

The World Health Organization and the United Nations Children’s Fund developed the Global Immunization Vision and Strategy (GIVS) 2006-2015. One of the set goals of GIVS for any country is to reach at least 90% national vaccination coverage and at least 80% vaccination coverage in every district or equivalent administrative unit [3].

In Yemen, the strategy for the national Expanded Program on Immunization (EPI) 2011-2015 was to reach 95% coverage at the national level, and not less than 80% for diphtheria, tetanus, and pertussis at the district level by 2015 [4]. However, a major demographic challenge for reaching this target is a very scattered population, with more than 130,000 population sites all over the country [5]. Although, the political crisis that started in 2011 with the Arab Spring had negatively affected the health system and accessibility to health services, including vaccination, the political situation stabilized after 2012. However, in March 2015, a major war broke out and left only 45% of health facilities functioning. Yemen, which had a fragile health care system before the onset of war, did not have the infrastructure to withstand such a catastrophe and is currently on the brink of collapse. In the past 2 years, continuous war has led to the destruction of health care facilities and has made access to the ones standing difficult for those in need. Before the war, Yemen had a relatively stable vaccination rate reaching 70% to 80% of the target population; however, this dropped remarkably after the war [6,7]. VPD such as measles, cholera, and diphtheria saw a sudden surge after the beginning of the war [7-11]. Polio importation in such a critical situation also remains a major threat not only for Yemen but also for its neighboring countries.

### Objective

This study aimed to determine the immunization coverage of the children under 1 year, during the 2015 war.

## Methods

A soft copy of data aggregated by EPI during 2015-2015 was obtained from EPI in Excel format, which collected data from all 23 governorates, 333 districts, and 3096 vaccination sites in Yemen. The data included the following variables: the total population, targeted children, and the number of vaccinated children by each vaccine type and governorate. The vaccination coverage was calculated at the national and governorate levels by dividing the number of actually vaccinated children by the estimated population of children under 1 year. The governorates were divided into confrontation and nonconfrontation governorates depending on the battle on the ground. Analysis was carried out using Epi Info 7.2 (CDC) and Excel. Data were described using graphs and percentages.

## Results

**Figure 1:**
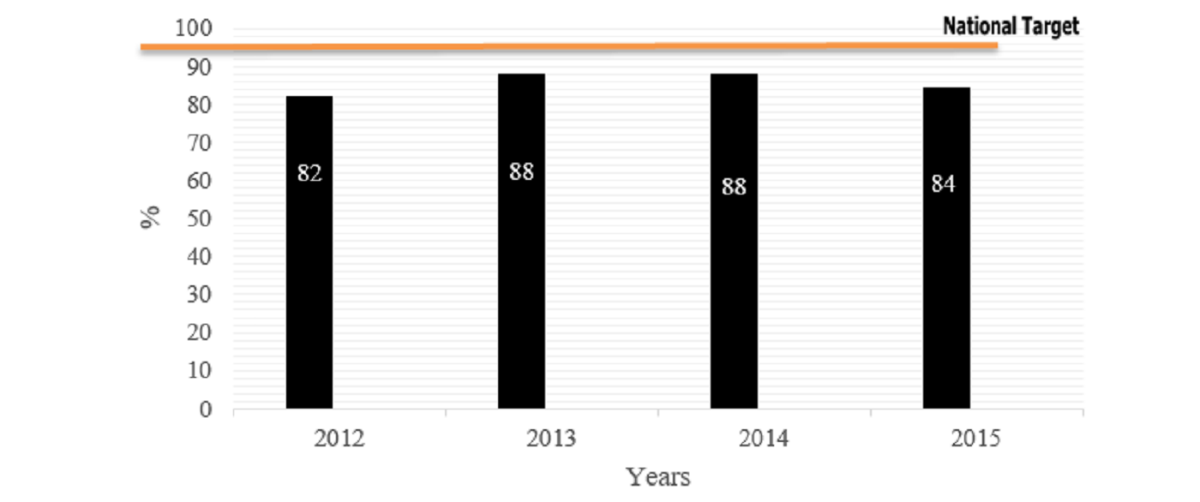
Immunization coverage for penta-3 vaccine compared to the national target, 2012-2015.

**Figure 2:**
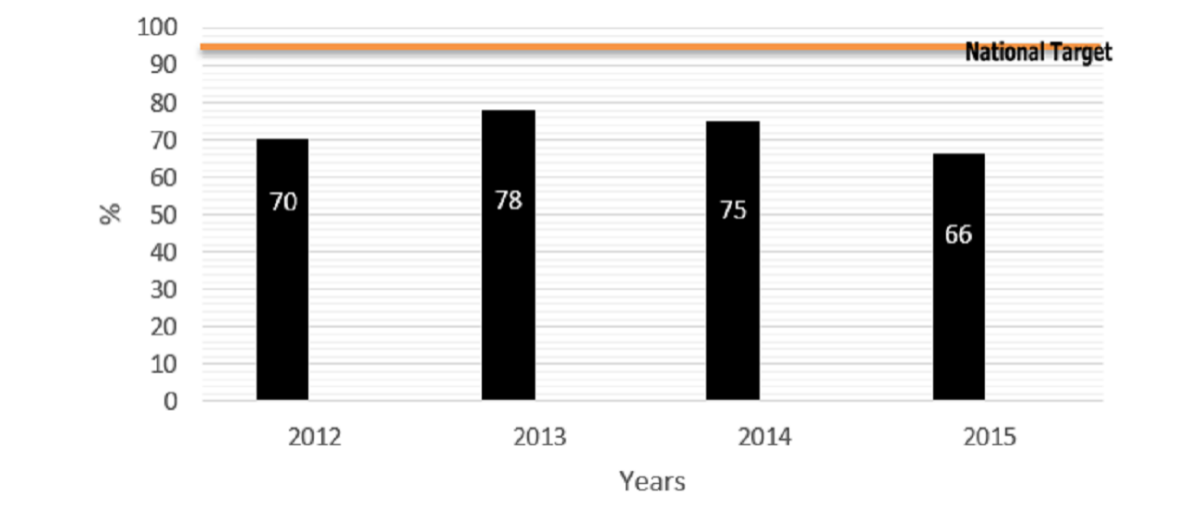
Immunization coverage for measles compared to the national target, 2012-2015.

The immunization coverage increased for penta-3 vaccine and measles coverage of vaccination (MCV) during 2012-2014, but coverage dropped in 2015. Compared with the national target, the coverage was still below the national target (Figures 1 and 2).

The first table in Multimedia Appendix 1 summarizes the immunization coverage by vaccine type for 2012-2015, which shows some increase in the coverage from 2012 to 2014 for all vaccines; however, there is a drop in the coverage in the year 2015 that witnessed the war (out of 3096 vaccination sites, 2577 sites reported of providing vaccination).

The second table in Multimedia Appendix 1 shows penta-3 vaccine coverage by governorates. There was an increase in the vaccination coverage in most governorates between 2011 and 2014, except for some governorates such as Al Jawf and Sa’dah. With the eruption of war in 2015, there was a marked drop in the vaccination coverage in some governorates such as Al Jawf, Sa’dah, Lahj, and Taiz. On the other hand, some governorates showed an increase in the coverage (eg, Ibb, Raymah, and Sana’a governorates).

## Discussion

### Principal Findings

Despite recent progress in vaccination coverage in Yemen, a very scattered population, with more than 130,000 population sites all over the country, poses a major challenge. Furthermore, the current political situation and insecurity have negatively affected the progress. Our findings showed a gradual increase in the coverage of penta-3 vaccine and MCV during 2012-2014, compared with the year 2011 that witnessed the political crises and the Arab Spring. Such an increase might be owing to the relative political stability and improvement of security after the uprising came to a successful conclusion. However, Al Jawf and Sa’dah governorates did not show such an increase as they continue to suffer from insecurity and fights, in addition to having sparse population with difficult access to vaccines and poor community awareness and wrong beliefs regarding immunization [12]. Furthermore, in all these years the coverage was still below the national target [4] and lower than the coverage in the neighboring countries, including Saudi Arabia (96%) and Oman (99%), but better than that in Somalia (65%) [13,14].

The coverage remarkably dropped in 2015 owing to the eruption of the war. Such a drop was more apparent in governorates that witnessed confrontations such as Sa’dah, Lahj, and Taiz. For example, Taiz governorate showed a drop in penta-3 vaccine coverage from (102,455/110,755) 92.51% in 2014 to (83,182/113,535) 73.27% in 2015 and Sa’dah from (17,511/26,779) 65.64% to (13,849/27,754) 49.90%. However, the coverage increased in Ibb and Raymah governorates that were less affected by the war and showed an increase in their population because of displacement from less secure and confrontation-prone governorates. For example, Raymah showed an increase in penta-3 vaccine coverage from (15,868/18,255) 86.92% to (19,871/18,810) 105.64% and Ibb from (93,106/99,919) 93.18% to (103,435/102,417) 100.99% in 2015.

### Conclusions

In conclusion, this analysis shows the marked negative impact of the 2015 war on immunization coverage, especially in the governorates that witnessed armed confrontations. This could put Yemen at more risk of VPD outbreaks and polio importation. Besides the ongoing efforts to stop the Yemeni war, strategies for more innovative vaccine delivery/provision and fulfilling the increasing demands are needed, especially in governorates with confrontations. Enhancing EPI performance through supportable investments in infrastructure that was destroyed by the war and providing decentralized funds are a prerequisite. Further studies to assess the effect of continuous war on vaccination coverage and to measure the resilience are recommended.

## Appendix

Multimedia Appendix 1 Immunization coverage (percentage) by vaccine type 2012-2015 and penta-3 coverage by governorates, 2012- 2015.

## Abbreviations

EPI: Expanded Program on Immunization
GIVS: Global Immunization Vision and Strategy
MCV: measles coverage of vaccination
VPD: vaccine-preventable diseases
