# Supplementary material for: The Impact of War in Yemen on Immunization Coverage of Children Under One Year of Age: Descriptive Study
Source: JMIR Public Health Surveill. 2019 Oct 23;5(4):e14461. doi: 10.2196/14461 (PMC6913770; doi:10.2196/14461)
Supplement: Multimedia Appendix 1 [file publichealth_v5i4e14461_app1.pdf]

Table 1. Immunization coverage (percentage) by vaccine type 2012-2015

Vaccine	2012	2013	2014	2015
BCG	64	74	73	49
Primary Polio	33	37	38	29
1st polio	89	94	94	89
2nd polio	84	90	89	86
3rd polio	82	88	88	84
4th polio	46	67	64	12
1st penta	89	94	94	89
2nd penta	84	90	89	86
3rd penta	82	88	88	84
1st pneumonia	88	94	93	89
2nd pneumonia	83	90	89	86
3rd pneumonia	82	88	88	84
1st Rota	35	79	79	72
2nd Rota	23	71	72	67
Measles	71	78	76	66
Vit. A	49	60	59	47

Table 2. Penta 3 coverage by governorates, 2012- 2015

**Governorate**		**2012**	**2013**	**2014**	**2015**
None - confrontation	**Ibb**	86	95	93	101*
	**Abyan**	77	89	86	85
	**Alamana**	81	91	87	73
	**Al Bayda'**	83	85	85	82
	**Hudaydah**	80	91	92	95
	**Mahwit**	81	85	91	92
	**Mahra**	71	67	68	71
	**Haja**	89	93	93	91
	**Coastal Hadramout**	88	87	86	83
	**Hadramout Wadi and Desert**	81	85	86	80
	**Dhamar**	94	96	94	97
	**Shabwah**	71	83	81	77
	**Sana'a**	80	94	97	99
	**Raymah**	84	85	87	106
confrontation	**Aden**	87	90	90	70
	**Amran**	78	85	89	86
	**Lahj**	90	96	96	75
	**Ma'rib**	81	87	89	76
	**Al Jwaf**	7	13	9	40
	**Taiz**	88	91	93	73
	**Sa`dah**	76	66	65	50
	**Al Dali'**	76	90	88	77

*increased due to displaced people from Taiz and other governorates
